# Supplementary material for: Radiocarbon and genomic evidence for the survival of Equus Sussemionus until the late Holocene
Source: eLife. 2022 May 11;11:e73346. doi: 10.7554/eLife.73346 (PMC9142152; doi:10.7554/eLife.73346)
Supplement: Supplementary file 1. — (a) Sample information. Dates are estimated from either calibrated radiocarbon dating (bold) or from the archaeological context. Sex is inferred from the ratio of depth of coverage found on the X chromosome and autosomes (F, female; M, male) (c), and the average depth of coverage when mapping against both of the horse and donkey reference genomes after rescaling and trimming are provided. (b) Calibrated radiocarbon measurement summary statistics and dating of five ancient horses sequenced in this study. Uncal BP dates were calibrated using OxCalOnline (https://c14.arch.ox.ac.uk/oxcal.html) with the IntCal20 calibration curve. (c) Sex information. The mean coverage of the autosomes and the X chromosome together with the ratio between them (F, female; M, male). (d) Comparative Genome Panel. (e) Mitochondrial sequences used in this study. (f) Variance explained by TreeMix models from 0 to 3 migration edges excluding transitions. Monotonic increase of the variance explained by the model stopped when considering more than 3 migration edges. (g) Inference of total migration rates (M) and migration proportions (p) using G-PhoCS (Gronau et al., 2011). A total of five models, including various possible migration bands, were considered. Models 1–4 include migration bands between E. ovodovi and other lineages, while model 5 contains all gene flow events identified in Jónsson et al., 2014. The migration bands with significant gene flow are highlighted in bold (these were defined as having a mean value of M > 3% and 95% credible interval not intercepting 0). They were combined to establish the final demographic model shown in Figure 3. (h). Migration rate estimates returned by G-PhoCS. The 95% credible intervals of four significant migration bands identified in (g) are shown. (i) Parameter estimates returned by G-PhoCS, considering models with and without migrations. The topology is in the form of (E. caballus, (E. ovodovi, ((E. a. somalicus, E. a. africanus), (E. kiang, E. hemi [file elife-73346-supp1.docx]

**Supplementary File 1a****.** Sample information. Dates are estimated from either calibrated radiocarbon dating (bold) or from the archaeological context. Sex is inferred from the ratio of depth-of-coverage found on the X chromosome and autosomes (F, female; M, Male) (Supplementary File 1c), and the average depth-of-coverage when mapping against both of the horse and donkey reference genomes after rescaling and trimming are provided.

|  |  |  |  |  | **Horse ref** | | **Donkey ref** | |
| --- | --- | --- | --- | --- | --- | --- | --- | --- |
| **Sample ID** | **Skeletal element** | **Sex** | **Age ( BP)** | **Location (all in China)** | **nuDNA Coverage** | **mtDNA Coverage** | **nuDNA Coverage** | **mtDNA Coverage** |
| BY01H | Teeth | M | **4,056-4,298** | Shatangbeiyuan, Ningxia Province | 0.0941 | 3.3321 | 0.1057 | 4.4170 |
| BY04H | Axis | M | 3,900-4,200 | Shatangbeiyuan, Ningxia Province | 0.0026 | 0.6637 | 0.0029 | 0.8098 |
| BY05H | Jaw | M | 3,900-4,200 | Shatangbeiyuan, Ningxia Province | 0.0025 | 0.6637 | 0.0027 | 0.8848 |
| HH01D | Premaxillary | F | 3,400-4,400 | Honghe, Heilongjiang Province | 0.0027 | 0.8527 | 0.0030 | 0.9061 |
| HH03H | Teeth | F | 3,400-4,400 | Honghe, Heilongjiang Province | 0.4109 | 19.4920 | 0.4617 | 25.017 |
| HH04D | Pastern | M | **4,222-4,476** | Honghe, Heilongjiang Province | 3.4784 | 370.5604 | 3.8608 | 490.836 |
| HH06D | Teeth | F | **4,227-4,481** | Honghe, Heilongjiang Province | 12.0068 | 251.7237 | 13.3974 | 319.697 |
| HH07H | Teeth | M | 3,400-4,400 | Honghe, Heilongjiang Province | 0.0483 | 1.2793 | 0.0541 | 1.5641 |
| HH13H | Radius | M | **3,477-3,637** | Honghe, Heilongjiang Province | 0.0546 | 8.9469 | 0.0600 | 11.7211 |
| HH14H | Scapula | M | 3,400-4,400 | Honghe, Heilongjiang Province | 0.9203 | 42.6442 | 1.0415 | 54.3286 |
| HH18H | Metacarpus | M | 3,400-4,400 | Honghe, Heilongjiang Province | 1.0386 | 8.0227 | 1.1410 | 10.7308 |
| HH19H | Radius | M | 3,400-4,400 | Honghe, Heilongjiang Province | 0.3448 | 4.7541 | 0.3869 | 6.1105 |
| HH20H | Scapula | F | 3,400-4,400 | Honghe, Heilongjiang Province | 0.0975 | 19.4459 | 0.1076 | 25.3521 |
| HH21H | Radius | F | 3,400-4,400 | Honghe, Heilongjiang Province | 0.1558 | 19.0956 | 0.1755 | 25.2176 |
| HH22H | Pastern | F | 3,400-4,400 | Honghe, Heilongjiang Province | 0.0197 | 3.9983 | 0.0216 | 5.0718 |
| HH26H | Calcaneus | M | 3,400-4,400 | Honghe, Heilongjiang Province | 0.0515 | 4.0265 | 0.0568 | 5.1053 |
| HH28H | Tibia | F | 3,400-4,400 | Honghe, Heilongjiang Province | 0.0028 | 0.2840 | 0.0032 | 0.4268 |
| HH29H | Humerus | M | 3,400-4,400 | Honghe, Heilongjiang Province | 0.0060 | 0.6803 | 0.0067 | 0.9427 |
| HH30H | Talus | M | 3,400-4,400 | Honghe, Heilongjiang Province | 0.0294 | 1.3584 | 0.0324 | 1.8471 |
| HH31H | Occipital | F | 3,400-4,400 | Honghe, Heilongjiang Province | 0.0543 | 0.7008 | 0.0618 | 0.8590 |
| HH32H | Pastern | F | 3,400-4,400 | Honghe, Heilongjiang Province | 0.0034 | 0.5498 | 0.0037 | 0.6731 |
| HH34H | Occipital | M | 3,400-4,400 | Honghe, Heilongjiang Province | 0.0091 | 0.2525 | 0.0101 | 0.3116 |
| HH39H | Humerus | F | 3,400-4,400 | Honghe, Heilongjiang Province | 0.0115 | 2.6107 | 0.0127 | 3.2649 |
| MZ103H | Tibia | F | 3,800-4,300 | Muzhuzhuliang, Shaanxi Province | 0.0018 | 0.2042 | 0.0020 | 0.2131 |
| MZ104H | Scapula | M | 3,800-4,300 | Muzhuzhuliang, Shaanxi Province | 0.0026 | 2.0642 | 0.0029 | 2.6481 |
| MZ105H | Humerus | M | **3,803-4,049** | Muzhuzhuliang, Shaanxi Province | 0.0577 | 5.1511 | 0.0642 | 6.6867 |

**Supplementary File 1b.** Calibrated radiocarbon measurement summary statistics and dating of 5 ancient horses sequenced in this study. Uncal BP dates were calibrated using OxCalOnline (<https://c14.arch.ox.ac.uk/oxcal.html>) with the IntCal20 calibration curve.

|  |  |  |  |  |  |  |  |  |  | **IntCal20** | | | | |
| --- | --- | --- | --- | --- | --- | --- | --- | --- | --- | --- | --- | --- | --- | --- |
|  |  |  |  |  |  |  |  |  |  | **calBCE** | | | |  |
| **Sample ID** | **Lab accession number** | **δ13C (‰)** | **δ15N (‰)** | **Fraction Modern Carbon** | **∆14C (‰)** | **14C age (BP)** | **C/N** | **%C** | **%N** | **From** | **To** | **Interval (%)** | **Average** | **Years ago (from 2021)** |
| BY01H | Beta - 503260 | -19.3 | 5.3 | 0.6278 ± 0.0023 | -377.37 ± 2.34 | 3740 ± 30 | 3.2 | 41.53 | 15.29 | -2277 | -2035 | 95.4 | -2156 | -4177 |
| HH04D | Beta - 463384 | -19.2 | 3.5 | 0.6200 ± 0.0023 | -385.00 ± 2.32 | 3840 ± 30 | 3.2 | 42.87 | 15.73 | -2455 | -2201 | 95.4 | -2328 | -4349 |
| HH06D | Beta - 500199 | -17.6 | 4.2 | 0.6185 ± 0.0023 | -386.61 ± 2.31 | 3860 ± 30 | 3.2 | 41.62 | 15.28 | -2460 | -2206 | 95.4 | -2333 | -4354 |
| HH13H | Beta - 500200 | -18.8 | 3.9 | 0.6656 ± 0.0025 | -339.86 ± 2.49 | 3270 ± 30 | 3.1 | 39.41 | 14.87 | -1616 | -1456 | 95.4 | -1536 | -3557 |
| MZ105H | Beta - 503259 | -15.8 | 4.2 | 0.6404 ± 0.0024 | -364.85 ± 2.39 | 3580 ± 30 | 3.2 | 41.56 | 15.06 | -2028 | -1782 | 95.4 | -1905 | -3926 |

**Supplementary File 1c.** Sex information. The mean coverage of the autosomes and the X chromosome together with the ratio between them (F, female; M, Male).

| **Sample ID** | **Cov_auto** | **Cov_X** | **auto/X** | **Sex** |
| --- | --- | --- | --- | --- |
| BY01H | 0.0997 | 0.0501 | 1.99 | M |
| BY04H | 0.0027 | 0.0013 | 2.11 | M |
| BY05H | 0.0026 | 0.0011 | 2.39 | M |
| HH01D | 0.0028 | 0.0025 | 1.11 | F |
| HH03H | 0.4241 | 0.4286 | 0,99 | F |
| HH04D | 3.6739 | 1.7827 | 2.06 | M |
| HH06D | 12.4089 | 12.0420 | 1.03 | F |
| HH07H | 0.0513 | 0.0269 | 1.91 | M |
| HH13H | 0.0577 | 0.0306 | 1.88 | M |
| HH14H | 0.9751 | 0.4954 | 1.97 | M |
| HH18H | 1.1012 | 0.5652 | 1.95 | M |
| HH19H | 0.3662 | 0.1720 | 2.13 | M |
| HH20H | 0.1004 | 0.1031 | 0.97 | F |
| HH21H | 0.1607 | 0.1583 | 1.02 | F |
| HH22H | 0.0201 | 0.0209 | 0.97 | F |
| HH26H | 0.0545 | 0.0255 | 2.14 | M |
| HH28H | 0.0029 | 0.0030 | 0.98 | F |
| HH29H | 0.0064 | 0.0034 | 1.87 | M |
| HH30H | 0.0311 | 0.0159 | 1.95 | M |
| HH31H | 0.0559 | 0.0579 | 0.97 | F |
| HH32H | 0.0035 | 0.0034 | 1.03 | F |
| HH34H | 0.0096 | 0.0049 | 1.96 | M |
| HH39H | 0.0119 | 0.0119 | 0.99 | F |
| MZ103H | 0.0018 | 0.0016 | 1.14 | F |
| MZ104H | 0.0028 | 0.0014 | 1.93 | M |
| MZ105H | 0.0611 | 0.0319 | 1.92 | M |

**Supplementary File 1d.** Comparative Genome Panel.

| **Sample ID** | **Raw Name/Abr.** | **Sex** | **Species** | **Accession** | **Source** | **nuDNA Coverage** |
| --- | --- | --- | --- | --- | --- | --- |
| AFR | Willy1 | M | *Equus asinus africanus* | SAMEA104569895 | ([Renaud et al., 2018](#_ENREF_11)) | 43.2 |
| BOE | BOE | F | *Equus burchellii boehmi* | SAMEA2797679 | ([Jonsson et al., 2014](#_ENREF_7)) | 17.4 |
| DON | Willy | M | *Equus asinus asinus* | SAMN02179859 | ([Orlando et al., 2013](#_ENREF_10)) | 10.5 |
| GRE | GRE | F | *Equus grevyi* | SAMEA3166710 | ([Jonsson et al., 2014](#_ENREF_7)) | 15.6 |
| HAR | HAR | F | *Equus zebra hartmannae* | SAMEA2802528 | ([Jonsson et al., 2014](#_ENREF_7)) | 16.3 |
| ONA | ONA | M | *Equus hemionus* | SAMEA2802530 | ([Jonsson et al., 2014](#_ENREF_7)) | 18.3 |
| HEM | Hulan sharag | M | *Equus hemionus* | SAMN03010637 | ([Huang et al., 2015](#_ENREF_6)) | 8.5 |
| KIA | KIA | F | *Equus kiang* | SAMEA2802529 | ([Jonsson et al., 2014](#_ENREF_7)) | 11.6 |
| QUA | QUA | F | *Equus burchellii quagga* | SAMEA3166709 | ([Jonsson et al., 2014](#_ENREF_7)) | 7.9 |
| SOM | SOM | F | *Equus asinus somalicus* | SAMEA2802531 | ([Jonsson et al., 2014](#_ENREF_7)) | 21.0 |
| TWI | Twilight | F | *Equus caballus* | SAMN02953672 | ([Kalbfleisch et al., 2018](#_ENREF_8)) | 33.8 |

**Supplementary File 1e.** Mitochondrial sequences used in this study.

| **Species** | **Age** | **Accession/ID** | **Source** |
| --- | --- | --- | --- |
| *Equus przewalskii* | modern | JN398402 | ([Achilli et al., 2012](#_ENREF_1)) |
| *Equus zebra hartmannae* | modern | JX312719 | ([Vilstrup et al., 2013](#_ENREF_12)) |
| *Equus burchellii chapmani* | modern | JX312721 | ([Vilstrup et al., 2013](#_ENREF_12)) |
| *Equus grevyi* | modern | JX312725 | ([Vilstrup et al., 2013](#_ENREF_12)) |
| *Equus hemionus onager* | modern | JX312730 | ([Vilstrup et al., 2013](#_ENREF_12)) |
| *Equus kiang* | modern | JX312732 | ([Vilstrup et al., 2013](#_ENREF_12)) |
| *Equus ovodovi* | 40,000 yBP | JX312734 | ([Vilstrup et al., 2013](#_ENREF_12)) |
| *Hippidion saldiasi* | 16,809-17,400 yBP | KM881671 | ([Der Sarkissian et al., 2015](#_ENREF_2)) |
| *Equus burchellii quagga* | 150 yBP | KM881680 | ([Jonsson et al., 2014](#_ENREF_7)) |
| *Equus asinus somalicus* | modern | KM881681 | ([Jonsson et al., 2014](#_ENREF_7)) |
| *Equus caballus* | 4,415-4,485 yBP | KT368725 | ([Librado et al., 2015](#_ENREF_9)) |
| *Equus caballus* | 43,962-42,044 yBP | KT757740 | ([Orlando et al., 2013](#_ENREF_10)) |
| *Equus caballus* | 16,850-15,994 yBP | KT757741 | ([Orlando et al., 2013](#_ENREF_10)) |
| *Equus ovodovi* | 32,000 yBP | KY114520 | ([Druzhkova et al., 2017](#_ENREF_3)) |
| *Haringtonhippus francisci* | 13,015-13,125 yBP | MF134655 | ([Heintzman et al., 2017](#_ENREF_5)) |
| *Equus caballus* | modern | X79547 | ([X. Xu & Arnason, 1994](#_ENREF_13)) |
| *Equus asinus asinus* | modern | X97337 | ([Xiufeng Xu, Gullberg, & Arnason, 1996](#_ENREF_14)) |
| *Equus ovodovi* | 12,596–12,770 yBP | ZDT9 | ([Yuan et al., 2019](#_ENREF_15)) |
| *Equus ovodovi* | 28,887–29,525 yBP | ZDT7 | ([Yuan et al., 2019](#_ENREF_15)) |
| *Equus ovodovi* | 38,848–40,201 yBP | ZDT4 | ([Yuan et al., 2019](#_ENREF_15)) |

**Supplementary File 1f.** Variance explained by TreeMix models from 0 to 3 migration edges excluding transitions. Monotonic increase of the variance explained by the model stopped when considering more than 3 migration edges.

| **Number of migrations** | **Variance**  **(horse reference)** | **Variance**  **(donkey reference)** |
| --- | --- | --- |
| 0 | 0.9892441 | 0.9830742 |
| 1 | 0.9903087 | 0.984263 |
| 2 | 0.9908188 | 0.9849425 |
| 3 | 0.9913539 | 0.9849345 |

**Supplementary File 1g.** Inference of total migration rates (*M*) and migration proportions (*p*) using G-PhoCS ([Gronau, Hubisz, Gulko, Danko, & Siepel, 2011](#_ENREF_4)). A total of 5 models, including various possible migration bands, were considered. Models 1 to 4 include migration bands between *E. ovodovi* and other lineages, while model 5 contains all gene flow events identified in ([Jonsson et al., 2014](#_ENREF_7)). The migration bands with significant gene flow were highlighted in bold (these were defined as having a mean value of *M* > 3% and 95% credible interval not intercepting 0). They were combined to establish the final demographic model shown in Fig 3.

|  | **Total migration rates (*M*)** | | | **Probability of migration (*p*)** | | |
| --- | --- | --- | --- | --- | --- | --- |
| **model1** | **mean** | **95% HPD Interval** | | **mean** | **95% HPD Interval** | |
|  |  | **lower** | **upper** |  | **lower** | **upper** |
| *E. a. somalicus* → *E. ovodovi* | 0.03% | 0.00% | 0.14% | 0.03% | 0.00% | 0.14% |
| *E. ovodovi* → *E. a. somalicus* | 0.11% | 0.02% | 0.22% | 0.11% | 0.02% | 0.22% |
| *E. a. africanus* → *E. ovodovi* | 0.04% | 0.00% | 0.18% | 0.04% | 0.00% | 0.18% |
| *E. ovodovi* → *E. a. africanus* | 0.01% | 0.00% | 0.03% | 0.01% | 0.00% | 0.03% |
| *E. kiang* → *E. ovodovi* | 0.10% | 0.00% | 0.48% | 0.10% | 0.00% | 0.48% |
| *E. ovodovi* →*E. kiang* | 0.04% | 0.00% | 0.16% | 0.04% | 0.00% | 0.16% |
| *E. hemionus* → *E. ovodovi* | 0.42% | 0.00% | 0.84% | 0.42% | 0.00% | 0.84% |
| *E. ovodovi* → *E. hemionus* | 0.01% | 0.00% | 0.08% | 0.01% | 0.00% | 0.08% |
| **model2** | **mean** | **95% HPD Interval** | | **mean** | **95% HPD Interval** | |
|  |  | **lower** | **upper** |  | **lower** | **upper** |
| Anc (Asian ass) → *E. ovodovi* | 0.08% | 0.00% | 0.38% | 0.08% | 0.00% | 0.38% |
| *E. ovodovi* → Anc (Asian ass) | 0.03% | 0.00% | 0.17% | 0.03% | 0.00% | 0.17% |
| Anc (African ass) → *E. ovodovi* | 0.01% | 0.00% | 0.04% | 0.01% | 0.00% | 0.04% |
| *E. ovodovi* → Anc (African ass) | 0.03% | 0.00% | 0.14% | 0.03% | 0.00% | 0.14% |
| **Anc (ass) → *E. ovodovi*** | 145.27% | 136.98% | 155.27% | 76.61% | 74.58% | 78.83% |
| ***E. ovodovi* → Anc (ass)** | 12.38% | 9.51% | 15.09% | 11.65% | 9.07% | 14.01% |
| **model3** | **mean** | **95% HPD Interval** | | **mean** | **95% HPD Interval** | |
|  |  | **lower** | **upper** |  | **lower** | **upper** |
| *E. b. boehmi* → *E. ovodovi* | 0.01% | 0.00% | 0.06% | 0.01% | 0.00% | 0.06% |
| *E. ovodovi* → *E. b. boehmi* | 0.06% | 0.00% | 0.13% | 0.06% | 0.00% | 0.13% |
| *E. grevyi* → *E. ovodovi* | 0.36% | 0.00% | 1.05% | 0.36% | 0.00% | 1.05% |
| *E. ovodovi* → *E. grevyi* | 0.37% | 0.00% | 0.66% | 0.37% | 0.00% | 0.66% |
| *E. z. hartmannae* → *E. ovodovi* | 0.51% | 0.00% | 1.02% | 0.51% | 0.00% | 1.02% |
| *E. ovodovi* → *E. z. hartmannae* | 0.19% | 0.01% | 0.41% | 0.19% | 0.01% | 0.40% |
| *E. b. quagga* → *E. ovodovi* | 0.61% | 0.25% | 1.05% | 0.61% | 0.25% | 1.04% |
| *E. ovodovi* → *E. b. quagga* | 0.35% | 0.11% | 0.66% | 0.35% | 0.11% | 0.66% |
| **model4** | **mean** | **95% HPD Interval** | | **mean** | **95% HPD Interval** | |
|  |  | **lower** | **upper** |  | **lower** | **upper** |
| Anc (*E. b. quagga*, *E. b. boehmi*) → *E. ovodovi* | 0.23% | 0.00% | 0.84% | 0.23% | 0.00% | 0.84% |
| *E. ovodovi* → Anc (*E. b. quagga*, *E. b. boehmi*) | 0.13% | 0.00% | 0.36% | 0.13% | 0.00% | 0.36% |
| Anc ((*E. b. quagga*, *E. b. boehmi*), *E. grevyi*) → *E. ovodovi* | 0.10% | 0.00% | 0.60% | 0.10% | 0.00% | 0.59% |
| *E. ovodovi* → *Anc* ((*E. b. quagga*, *E. b. boehmi*), *E. grevyi*) | 1.45% | 0.36% | 2.65% | 1.44% | 0.36% | 2.62% |
| **Anc (zebra) → *E. ovodovi*** | 40.48% | 32.30% | 50.78% | 33.29% | 27.60% | 39.82% |
| *E. ovodovi* → Anc (zebra) | 2.14% | 0.00% | 4.83% | 2.12% | 0.00% | 4.72% |
| **model5** | **mean** | **95% HPD Interval** | | **mean** | **95% HPD Interval** | |
|  |  | **lower** | **upper** |  | **lower** | **upper** |
| *E. a. africanus*→ *E. z. hartmannae* | 0.11% | 0.02% | 0.28% | 0.11% | 0.02% | 0.28% |
| *E. z. hartmannae* → *E. a. africanus* | 0.00% | 0.00% | 0.01% | 0.00% | 0.00% | 0.01% |
| *E. a. africanus* → *E. kiang* | 0.08% | 0.00% | 0.32% | 0.08% | 0.00% | 0.31% |
| *E. kiang*→ *E. a. africanus* | 0.00% | 0.00% | 0.04% | 0.00% | 0.00% | 0.04% |
| *E. b. boehmi* → *E. z. hartmannae* | 0.79% | 0.38% | 1.28% | 0.79% | 0.38% | 1.27% |
| *E. z. hartmannae* → *E. b. boehmi* | 0.19% | 0.02% | 0.40% | 0.18% | 0.02% | 0.40% |
| *E. a. africanus* → *E. z. hartmannae* | 0.01% | 0.02% | 0.28% | 0.01% | 0.02% | 0.28% |
| *E. z. hartmannae* → *E. a. africanus* | 0.00% | 0.00% | 0.01% | 0.00% | 0.00% | 0.01% |
| ***E. caballus* → Anc (*E. ovodovi*, *noncaballine*)** | 3.21% | 1.59% | 5.43% | 3.16% | 1.57% | 5.28% |

**Supplementary File 1h.** Migration rate estimates returned by G-PhoCS. The 95% credible intervals of 4 significant migration bands identified in Supplementary File 1g are shown.

|  | **Total Migration rate (*M*)** | | | **Probability of gene flow (*p*)** | | |
| --- | --- | --- | --- | --- | --- | --- |
|  | **mean** | **95% HPD Interval** | | **mean** | **95% HPD Interval** | |
|  |  | **lower** | **upper** |  | **lower** | **upper** |
| Anc (ass) → *E. ovodovi* | 111.8% | 91.3% | 126.9% | 67.3% | 59.9% | 71.9% |
| *E. ovodovi* → Anc (ass) | 13.5% | 8.7% | 17.4% | 12.6% | 8.4% | 16.0% |
| Anc (zebra) → *E. ovodovi* | 23.5% | 16.8% | 30.0% | 20.9% | 15.4% | 25.9% |
| *E. caballus* → Anc (*E. ovodovi*, noncaballine) | 5.4% | 2.2% | 9.2% | 5.3% | 2.2% | 8.8% |

**Supplementary File 1i.** Parameter estimates returned by G-PhoCS, considering models with and without migrations. The topology is in the form of (*E. caballus*, (*E. ovodovi*, ((*E. a. somalicus*, *E. a. africanus*), (*E. kiang*, *E. hemionus*)), (((*E. b. quagga*, *E. b. boehmi*), *E. grevyi*), *E. z. hartmannae*)))). Divergence time and population size are estimated by the 95% Bayesian credible interval using total 15,324 candidate ‘neutral’ loci, considering the sequence data aligned against the horse reference genome. The migration model contains the 4 significant migration bands estimated and provided in Supplementary File 1h**.**

|  | **no migration model** | | **migration model** | |
| --- | --- | --- | --- | --- |
| **Divergence time (yBP)** | **95% HPD Interval** | | **95% HPD Interval** | |
|  | **lower** | **upper** | **lower** | **upper** |
| Anc (African ass) | 252,306 | 310,695 | 235,657 | 282,734 |
| Anc (Asian ass) | 285,972 | 360,737 | 269,481 | 329,307 |
| Anc (ass) | 1,665,346 | 1,882,612 | 1,564,426 | 1,747,477 |
| Anc (*E. b. quagga*, *E. b. boehmi*) | 321,104 | 414,009 | 300,821 | 378,570 |
| Anc ((*E. b. quagga*, *E. b. boehmi*), *E. grevyi*) | 1,185,914 | 1,380,321 | 1,139,555 | 1,296,381 |
| Anc (zebra) | 1,624,042 | 1,800,879 | 1,459,985 | 1,616,102 |
| Anc (noncaballine) | 2,076,451 | 2,269,748 | 1,957,110 | 2,080,826 |
| Anc (*E. ovodovi*, noncaballine) | 2,081,351 | 2,274,694 | 2,365,255 | 2,701,784 |
| Root | 4,000,000 | 4,500,000 | 4,000,000 | 4,500,000 |
| **Effective population size** | **95% HPD Interval** | | **95% HPD Interval** | |
|  | **lower** | **upper** | **lower** | **upper** |
| *E. caballus* | 37,034 | 42,308 | 35,101 | 38,920 |
| *E. ovodovi* | 61,823 | 70,184 | 53,685 | 59,206 |
| *E. a. africanus* | 27,474 | 34,261 | 25,649 | 31,214 |
| *E. a. somalicus* | 13,863 | 17,028 | 12,940 | 15,489 |
| *E. kiang* | 17,888 | 22,270 | 16,843 | 20,352 |
| *E. hemionus* | 41,726 | 52,823 | 39,362 | 48,225 |
| *E. b. boehmi* | 203,370 | 312,624 | 191,508 | 285,776 |
| *E. grevyi* | 34,458 | 39,694 | 32,756 | 36,664 |
| *E. z. hartmannae* | 31,606 | 36,223 | 29,505 | 32,920 |
| *E. b. quagga* | 23,366 | 29,875 | 21,883 | 27,269 |
| Anc (African ass) | 39,995 | 46,518 | 37,670 | 42,828 |
| Anc (Asian ass) | 110,439 | 129,487 | 103,442 | 118,529 |
| Anc (ass) | 87,615 | 136,065 | 45,681 | 92,943 |
| Anc (*E. b. quagga*, *E. b. boehmi*) | 66,458 | 83,147 | 63,546 | 77,430 |
| Anc ((*E. b. quagga*, *E. b. boehmi*), *E. grevyi*) | 176,431 | 295,420 | 137,702 | 248,569 |
| Anc (zebra) | 25,928 | 33,365 | 31,727 | 45,580 |
| Anc (noncaballine) | 286 | 2,252 | 15,011 | 20,115 |
| Anc (*E. ovodovi*, noncaballine) | 38,662 | 44,396 | 41,304 | 50,628 |
| Root | 163,812 | 189,460 | 140,752 | 161,152 |

**Supplementary File 1j.** The tip dates (average calibrated radiocarbon dates or dates were estimated from the archaeological context) for sample ages in BEAST analyses.

| **Sample ID** | **Species** | **Date ( BP)** |
| --- | --- | --- |
| BY01H | This study | 4,177 |
| HH03H | This study | 3,900 |
| HH04D | This study | 4,349 |
| HH06D | This study | 4,354 |
| HH07H | This study | 3,900 |
| HH13H | This study | 3,557 |
| HH14H | This study | 3,900 |
| HH18H | This study | 3,900 |
| HH19H | This study | 3,900 |
| HH20H | This study | 3,900 |
| HH21H | This study | 3,900 |
| HH22H | This study | 3,900 |
| HH26H | This study | 3,900 |
| HH30H | This study | 3,900 |
| HH39H | This study | 3,900 |
| MZ104H | This study | 4,050 |
| MZ105H | This study | 3,926 |
| X97337 | *Equus asinus asinus* | 0 |
| KM881681 | *Equus asinus somalicus* | 0 |
| JX312721 | *Equus burchellii chapmani* | 0 |
| KM881680 | *Equus burchellii quagga* | 150 |
| KT368725 | *Equus caballus* | 4,450 |
| KT757740 | *Equus caballus* | 43,000 |
| KT757741 | *Equus caballus* | 16,400 |
| X79547 | *Equus caballus* | 0 |
| JX312725 | *Equus grevyi* | 0 |
| JX312730 | *Equus hemionus onager* | 0 |
| JX312732 | *Equus kiang* | 0 |
| JX312734 | *Equus ovodovi* | 40,000 |
| KY114520 | *Equus ovodovi* | 32,000 |
| ZDT4 | *Equus ovodovi* | 39,525 |
| ZDT7 | *Equus ovodovi* | 29,206 |
| ZDT9 | *Equus ovodovi* | 12,683 |
| JN398402 | *Equus przewalskii* | 100 |
| JX312719 | *Equus zebra hartmannae* | 0 |
| MF134655 | *Haringtonhippus francisci* | 13,070 |
| KM881671 | *Hippidion saldiasi* | 17,105 |

ample ages**References**

Achilli, A., Olivieri, A., Soares, P., Lancioni, H., Hooshiar Kashani, B., Perego, U. A., Nergadze, S. G., Carossa, V., Santagostino, M., Capomaccio, S., Felicetti, M., Al-Achkar, W., Penedo, M. C., Verini-Supplizi, A., Houshmand, M., Woodward, S. R., Semino, O., Silvestrelli, M., Giulotto, E., Pereira, L., Bandelt, H. J., & Torroni, A. (2012). Mitochondrial genomes from modern horses reveal the major haplogroups that underwent domestication. *Proc Natl Acad Sci U S A, 109*(7), 2449-2454. doi:10.1073/pnas.1111637109

Der Sarkissian, C., Vilstrup, J. T., Schubert, M., Seguin-Orlando, A., Eme, D., Weinstock, J., Alberdi, M. T., Martin, F., Lopez, P. M., Prado, J. L., Prieto, A., Douady, C. J., Stafford, T. W., Willerslev, E., & Orlando, L. (2015). Mitochondrial genomes reveal the extinct Hippidion as an outgroup to all living equids. *Biol Lett, 11*(3). doi:10.1098/rsbl.2014.1058

Druzhkova, Anna S., Makunin, Alexey I., Vorobieva, Nadezhda V., Vasiliev, Sergey K., Ovodov, Nikolai D., Shunkov, Mikhail V., Trifonov, Vladimir A., & Graphodatsky, Alexander S. (2017). Complete mitochondrial genome of an extinct Equus (Sussemionus) ovodovi specimen from Denisova cave (Altai, Russia). *Mitochondrial DNA Part B, 2*(1), 79-81. doi:10.1080/23802359.2017.1285209

Gronau, I., Hubisz, M. J., Gulko, B., Danko, C. G., & Siepel, A. (2011). Bayesian inference of ancient human demography from individual genome sequences. *Nat Genet, 43*(10), 1031-1034. doi:10.1038/ng.937

Heintzman, P. D., Zazula, G. D., MacPhee, R., Scott, E., Cahill, J. A., McHorse, B. K., Kapp, J. D., Stiller, M., Wooller, M. J., Orlando, L., Southon, J., Froese, D. G., & Shapiro, B. (2017). A new genus of horse from Pleistocene North America. *Elife, 6*. doi:10.7554/eLife.29944

Huang, J., Zhao, Y., Bai, D., Shiraigol, W., Li, B., Yang, L., Wu, J., Bao, W., Ren, X., Jin, B., Zhao, Q., Li, A., Bao, S., Bao, W., Xing, Z., An, A., Gao, Y., Wei, R., Bao, Y., Bao, T., Han, H., Bai, H., Bao, Y., Zhang, Y., Daidiikhuu, D., Zhao, W., Liu, S., Ding, J., Ye, W., Ding, F., Sun, Z., Shi, Y., Zhang, Y., Meng, H., & Dugarjaviin, M. (2015). Donkey genome and insight into the imprinting of fast karyotype evolution. *Sci Rep, 5*, 14106. doi:10.1038/srep14106

Jonsson, H., Schubert, M., Seguin-Orlando, A., Ginolhac, A., Petersen, L., Fumagalli, M., Albrechtsen, A., Petersen, B., Korneliussen, T. S., Vilstrup, J. T., Lear, T., Myka, J. L., Lundquist, J., Miller, D. C., Alfarhan, A. H., Alquraishi, S. A., Al-Rasheid, K. A., Stagegaard, J., Strauss, G., Bertelsen, M. F., Sicheritz-Ponten, T., Antczak, D. F., Bailey, E., Nielsen, R., Willerslev, E., & Orlando, L. (2014). Speciation with gene flow in equids despite extensive chromosomal plasticity. *Proc Natl Acad Sci U S A, 111*(52), 18655-18660. doi:10.1073/pnas.1412627111

Kalbfleisch, Theodore S., Rice, Edward S., DePriest, Michael S., Walenz, Brian P., Hestand, Matthew S., Vermeesch, Joris R., O′Connell, Brendan L., Fiddes, Ian T., Vershinina, Alisa O., Saremi, Nedda F., Petersen, Jessica L., Finno, Carrie J., Bellone, Rebecca R., McCue, Molly E., Brooks, Samantha A., Bailey, Ernest, Orlando, Ludovic, Green, Richard E., Miller, Donald C., Antczak, Douglas F., & MacLeod, James N. (2018). Improved reference genome for the domestic horse increases assembly contiguity and composition. *Communications Biology, 1*(1), 197. doi:10.1038/s42003-018-0199-z

Librado, P., Der Sarkissian, C., Ermini, L., Schubert, M., Jonsson, H., Albrechtsen, A., Fumagalli, M., Yang, M. A., Gamba, C., Seguin-Orlando, A., Mortensen, C. D., Petersen, B., Hoover, C. A., Lorente-Galdos, B., Nedoluzhko, A., Boulygina, E., Tsygankova, S., Neuditschko, M., Jagannathan, V., Theves, C., Alfarhan, A. H., Alquraishi, S. A., Al-Rasheid, K. A., Sicheritz-Ponten, T., Popov, R., Grigoriev, S., Alekseev, A. N., Rubin, E. M., McCue, M., Rieder, S., Leeb, T., Tikhonov, A., Crubezy, E., Slatkin, M., Marques-Bonet, T., Nielsen, R., Willerslev, E., Kantanen, J., Prokhortchouk, E., & Orlando, L. (2015). Tracking the origins of Yakutian horses and the genetic basis for their fast adaptation to subarctic environments. *Proc Natl Acad Sci U S A, 112*(50), E6889-6897. doi:10.1073/pnas.1513696112

Orlando, L., Ginolhac, A., Zhang, G., Froese, D., Albrechtsen, A., Stiller, M., Schubert, M., Cappellini, E., Petersen, B., Moltke, I., Johnson, P. L., Fumagalli, M., Vilstrup, J. T., Raghavan, M., Korneliussen, T., Malaspinas, A. S., Vogt, J., Szklarczyk, D., Kelstrup, C. D., Vinther, J., Dolocan, A., Stenderup, J., Velazquez, A. M., Cahill, J., Rasmussen, M., Wang, X., Min, J., Zazula, G. D., Seguin-Orlando, A., Mortensen, C., Magnussen, K., Thompson, J. F., Weinstock, J., Gregersen, K., Roed, K. H., Eisenmann, V., Rubin, C. J., Miller, D. C., Antczak, D. F., Bertelsen, M. F., Brunak, S., Al-Rasheid, K. A., Ryder, O., Andersson, L., Mundy, J., Krogh, A., Gilbert, M. T., Kjaer, K., Sicheritz-Ponten, T., Jensen, L. J., Olsen, J. V., Hofreiter, M., Nielsen, R., Shapiro, B., Wang, J., & Willerslev, E. (2013). Recalibrating Equus evolution using the genome sequence of an early Middle Pleistocene horse. *Nature, 499*(7456), 74-78. doi:10.1038/nature12323

Renaud, G., Petersen, B., Seguin-Orlando, A., Bertelsen, M. F., Waller, A., Newton, R., Paillot, R., Bryant, N., Vaudin, M., Librado, P., & Orlando, L. (2018). Improved de novo genomic assembly for the domestic donkey. *Sci Adv, 4*(4), eaaq0392. doi:10.1126/sciadv.aaq0392

Vilstrup, J. T., Seguin-Orlando, A., Stiller, M., Ginolhac, A., Raghavan, M., Nielsen, S. C., Weinstock, J., Froese, D., Vasiliev, S. K., Ovodov, N. D., Clary, J., Helgen, K. M., Fleischer, R. C., Cooper, A., Shapiro, B., & Orlando, L. (2013). Mitochondrial phylogenomics of modern and ancient equids. *PLoS One, 8*(2), e55950. doi:10.1371/journal.pone.0055950

Xu, X., & Arnason, U. (1994). The complete mitochondrial DNA sequence of the horse, Equus caballus: extensive heteroplasmy of the control region. *Gene, 148*(2), 357-362.

Xu, Xiufeng, Gullberg, Anette, & Arnason, Ulfur. (1996). The complete mitochondrial DNA (mtDNA) of the donkey and mtDNA comparisons among four closely related mammalian species-pairs. *Journal of Molecular Evolution, 43*(5), 438-446. doi:10.1007/BF02337515

Yuan, Jun-Xia, Hou, Xin-Dong, Barlow, Axel, Preick, Michaela, Taron, Ulrike H., Alberti, Federica, Basler, Nikolas, Deng, Tao, Lai, Xu-Long, Hofreiter, Michael, & Sheng, Gui-Lian. (2019). Molecular identification of late and terminal Pleistocene Equus ovodovi from northeastern China. *PLoS One, 14*(5), e0216883. doi:10.1371/journal.pone.0216883
