# Supplementary material for: Radiocarbon and genomic evidence for the survival of Equus Sussemionus until the late Holocene
Source: eLife. 2022 May 11;11:e73346. doi: 10.7554/eLife.73346 (PMC9142152; doi:10.7554/eLife.73346)
Supplement: Transparent reporting form [file elife-73346-transrepform1.docx]

***eLife’s* transparent reporting form**

We encourage authors to provide detailed information *within their submission* to facilitate the interpretation and replication of experiments. Authors can upload supporting documentation to indicate the use of appropriate reporting guidelines for health-related research (see [EQUATOR Network](http://www.equator-network.org/%20)), life science research (see the [BioSharing Information Resource](https://biosharing.org/)), or the [ARRIVE guidelines](http://www.plosbiology.org/article/info:doi/10.1371/journal.pbio.1000412) for reporting work involving animal research. Where applicable, authors should refer to any relevant reporting standards documents in this form.

If you have any questions, please consult our Journal Policies and/or contact us: [editorial@elifesciences.org](mailto:editorial@elifesciences.org).

**Sample-size estimation**

- You should state whether an appropriate sample size was computed when the study was being designed
- You should state the statistical method of sample size computation and any required assumptions
- If no explicit power analysis was used, you should describe how you decided what sample (replicate) size (number) to use

Please outline where this information can be found within the submission (e.g., sections or figure legends), or explain why this information doesn’t apply to your submission:

Sample size estimation can be found:

- Main manuscript:

Pag. 7, Archaeological samples and sequencing data;

Pag. 9, Taxonomic status.

- Supplementary material: Supplementary File 1a, d, e.

**Replicates**

- You should report how often each experiment was performed
- You should include a definition of biological versus technical replication
- The data obtained should be provided and sufficient information should be provided to indicate the number of independent biological and/or technical replicates
- If you encountered any outliers, you should describe how these were handled
- Criteria for exclusion/inclusion of data should be clearly stated
- High-throughput sequence data should be uploaded before submission, with a private link for reviewers provided (these are available from both GEO and ArrayExpress)

Please outline where this information can be found within the submission (e.g., sections or figure legends), or explain why this information doesn’t apply to your submission:

- Main manuscript, section Materials and methods:

- Morphometric analysis: pag. 7, Archaeological samples and sequencing data

- Ancient DNA experiment and bioinformatics processing: pag. 26, Genome Sequencing; pag. 29, Data Processing

- Data deposition: All sequencing data generated in this study have been deposited to the European Nucleotide Archive (https://www.ebi.ac.uk/ena/browser/home) under the accession number PRJEB44527.

**Statistical reporting**

- Statistical analysis methods should be described and justified
- Raw data should be presented in figures whenever informative to do so (typically when N per group is less than 10)
- For each experiment, you should identify the statistical tests used, exact values of N, definitions of center, methods of multiple test correction, and dispersion and precision measures (e.g., mean, median, SD, SEM, confidence intervals; and, for the major substantive results, a measure of effect size (e.g., Pearson's r, Cohen's d)
- Report exact p-values wherever possible alongside the summary statistics and 95% confidence intervals. These should be reported for all key questions and not only when the p-value is less than 0.05.

Please outline where this information can be found within the submission (e.g., sections or figure legends), or explain why this information doesn’t apply to your submission:

Main manuscript, section Materials and methods:

- Ancient DNA bioinformatic processing: pag. 26, Genome Sequencing

- Phylogenetic and Demographic Analysis:

Pag. 29, Data Processing

(For large datasets, or papers with a very large number of statistical tests, you may upload a single table file with tests, Ns, etc., with reference to sections in the manuscript.)

**Group allocation**

- Indicate how samples were allocated into experimental groups (in the case of clinical studies, please specify allocation to treatment method); if randomization was used, please also state if restricted randomization was applied
- Indicate if masking was used during group allocation, data collection and/or data analysis

Please outline where this information can be found within the submission (e.g., sections or figure legends), or explain why this information doesn’t apply to your submission:

Data collection:

- Main manuscript:

Figure 1;

Pag. 7, Archaeological samples and sequencing data; Pag. 9, Taxonomic status.

- Material and methods:

Pag. 29, Data Processing

**Additional data files (“source data”)**

- We encourage you to upload relevant additional data files, such as numerical data that are represented as a graph in a figure, or as a summary table
- Where provided, these should be in the most useful format, and they can be uploaded as “Source data” files linked to a main figure or table
- Include model definition files including the full list of parameters used
- Include code used for data analysis (e.g., R, MatLab)
- Avoid stating that data files are “available upon request”

Please indicate the figures or tables for which source data files have been provided:

Supplementary tables:
**Supplementary File 1a.** Sample information.

**Supplementary File 1b.** Calibrated radiocarbon measurement summary statistics and dating of 5 ancient horses sequenced in this study.

**Supplementary File 1c.** Sex information.

**Supplementary File 1d.** Comparative Genome Panel.

**Supplementary File 1e.** Mitochondrial sequences used in this study.

**Supplementary File 1f.** Variance explained by TreeMix models from 0 to 3 migration edges excluding transitions.

**Supplementary File 1g.** Inference of total migration rates (*M*) and migration proportions (*p*) using G-PhoCS.

**Supplementary File 1h.** Migration rate estimates returned by G-PhoCS.

**Supplementary File 1i.** Parameter estimates returned by G-PhoCS, considering models with and without migrations.

**Supplementary File 1j.** The tip dates (average calibrated radiocarbon dates or dates were estimated from the archaeological context) for sample ages in BEAST analyses.
